# Acknowledgement to Reviewers of *Geriatrics* in 2017

**DOI:** 10.3390/geriatrics3010002

**Published:** 2018-01-15

**Authors:** Geriatrics Editorial Office

**Affiliations:** MDPI AG, St. Alban-Anlage 66, 4052 Basel, Switzerland

Peer review is an essential part in the publication process, ensuring that *Geriatrics* maintains high quality standards for its published papers. In 2017, a total of 37 papers were published in the Geriatrics. Thanks to the cooperation of our reviewers, the median time to first decision was 26 days and the median time to publication was 55 days. The editors would like to express their sincere gratitude to the following reviewers for their time and dedication in 2017:
Anton, SteveLopez-Valcarcel, Beatriz GonzalezArba, FrancescoMattace-Raso, Francesco U. S.Atti, Anna RitaMinetto, Marco AlessandroBailey, CatherineMinicozzi, PamelaBerggren, IngelaMohler, JaneBoehm, Leanne M.Morandi, AlessandroBright, StephenMorino, KatsutaroBristowe, KatherineMorís, CésarBruins Slot, Karsten M. H.Mukaetova-Ladinska, ElizabetaBrzoska, PatrickMüller, MartijnBucci, MarcoNemeth, LynneBungum, TimothyPanza, FrancescoBurke, James F.Pekmezovic, TatjanaCauli, OmarPerry, Christina J.Cerejeira, JoaquimPowers, James S.Chung, Hsin-FangProcter-Gray, ElizabethCollard, Rose M.Putrino, DavidCornish, StephenQuinn, TerryDe Geest, BartRamani, Narayanaswamy VenketasubramanianDonini, LorenzoRay, Robin A.Elk, RonitRidley, Nicole J.Facal, DavidRouten, Ash C.Firmino, HoracioSantarpino, GiuseppeFulford, JonathanScalzo, FabienGallelli, LucaScharlach, AndrewGao, QiSearle, Samuel D.Gardner, Raquel C.Silverman, Michael J.Gauthier, SergeSingh, InderpalGeethakumari, Praveen RamakrishnanAl Snih, SohamGriebling, Tomas L.Stefani, AmbraGrimm, Marcus O. W.Sund-Levander, MärtaHaboubi, NadimSuñer-Soler, RosaHaider, LukasTang, Meng-ChiHaugland, TrudeTenore, GiancarloHeinz, MelindaThomas, ChristineHinyard, Leslie J.Tomata, YasutakeHolt, Tim A.Trabace, LuigiaHosie, AnnmarieTremolada, MartaHsiung, Ging Yuek RobinTroutman-Jordan, MeredithHsu, Hung-YiVaccaro, Joan A.Ihle, AndreasVaismoradi, MojtabaJacova, ClaudiaVieira, Edgar RamosJay, Sarah M.Visvanathan, AkilaJones, Richard N.Volaklis, Konstantinos A.Katja, KokkoVuksanovic, VesnaKelly, JanetWalters, MatthewKotronoulas, GrigoriosWatanabe, EiichiKratzke, CindyWerhagen, LarsLa Favor, Justin D.Whitley, EliseLauretani, FulvioWilliams, ElizabethLawrenson, RossWinberg, CeciliaLee, Sung JinYang, Jenq-LinLewis, Jordan P.Ye, JuanLim, Fidelindo A.Young, MichaelLlull, LauraYu, Tsung-HsienLodovici, Maura


